# Biologic medicine inclusion in 138 national essential medicines lists

**DOI:** 10.1186/s12969-021-00608-z

**Published:** 2021-09-06

**Authors:** Raphaël Kraus, Rae S. M. Yeung, Nav Persaud

**Affiliations:** 1 Department of Pediatrics, University of Toronto, Toronto, Canada; 2 Division of Rheumatology, Hospital for Sick Children, Toronto, Canada; 3 Departments of Pediatrics, Immunology and Medical Science, University of Toronto, Toronto, Canada; 4 Department of Family and Community Medicine, St. Michael’s Hospital, University of Toronto, Toronto, Canada

**Keywords:** World Health Organization, Essential medicines lists, Disease-modifying antirheumatic drugs (DMARDs), Biologics, Rare disease, Pediatrics

## Abstract

**Background:**

Essential medicines lists (EMLs) are intended to reflect the priority health care needs of populations. We hypothesized that biologic disease-modifying antirheumatic drugs (DMARDs) are underrepresented relative to conventional DMARDs in existing national EMLs. We aimed to survey the extent to which biologic DMARDs are included in EMLs, to determine country characteristics contributing to their inclusion or absence, and to contrast this with conventional DMARD therapies.

**Methods:**

We searched 138 national EMLs for 10 conventional and 14 biologic DMARDs used in the treatment of childhood rheumatologic diseases. Via regression modelling, we determined country characteristics accounting for differences in medicine inclusion between national EMLs.

**Results:**

Eleven countries (7.97%) included all 10 conventional DMARDs, 115 (83.33%) ≥5, and all countries listed at least one. Gross domestic product (GDP) per capita was associated with the total number of conventional DMARDs included (β_1_1.02 [95% CI 0.39, 1.66]; *P* = 0.00279). Among biologic DMARDs, 3 countries (2.2%) listed ≥10, 15 (10.9%) listed ≥5, and 47 (34.1%) listed at least one. Ninety-one (65.9%) of countries listed no biologic DMARDs. European region (β_1_ 1.30 [95% CI 0.08, 2.52]; *P* = 0.0367), life expectancy (β_1_–0.70 [95% CI -1.22, − 0.18]; *P* = 0.0085), health expenditure per capita (β_1_ 1.83 [95% CI 1.24, 2.42]; *P* < 0.001), and conventional DMARDs listed (β_1_ 0.70 [95% CI 0.33, 1.07]; *P* < 0.001) were associated with the total number of biologic DMARDs included.

**Conclusion:**

Biologic DMARDs are excluded from most national EMLs. By comparison, conventional DMARDs are widely included. Countries with higher health spending and longer life expectancy are more likely to list biologics.

**Supplementary Information:**

The online version contains supplementary material available at 10.1186/s12969-021-00608-z.

## Background

Rare diseases, by definition affecting small numbers of people relative to the general population (varying thresholds of maximal prevalence range from 5 to 76 per 100,000) and associated with specific issues relating to their rarity, represent an ever-growing subset of illness globally [1–3]. Recently, 6172 unique rare diseases were identified with an pooled global point prevalence of 3.5–5.9%, translating to 263–446 million affected persons worldwide [3]. More than half of rare diseases manifest in childhood with potentially disabling or even fatal consequence [2, 3]. All pediatric-onset rheumatologic conditions can be considered rare. Juvenile idiopathic arthritis (JIA), the most common rheumatologic disease in children, has a pooled prevalence of 45 per 100,000 [4, 5]. We employ JIA as a prototype for childhood rheumatologic disease, which broadly encompasses JIA, systemic lupus erythematosus (SLE), Sjögren syndrome, idiopathic inflammatory myopathies (namely juvenile dermatomyositis, or JDM), systemic and localized sclerodermas, systemic vasculitides, sarcoidosis, and autoinflammatory syndromes (among others).

Historically, conventional disease-modifying antirheumatic drugs (DMARDs) have provided the basis for therapy of pediatric systemic inflammatory disease. The advent of targeted biologic DMARD therapies has spurred a paradigm shift in the disease outcomes, patient experience, and prognosis of JIA and other rheumatologic conditions. Outcomes have improved dramatically resulting in increased survival and quality of life [6]. Additionally, biologic DMARD therapies—although costly—may be cost-effective in childhood rheumatologic disease: tumor necrosis factor (TNF) inhibitors for the treatment of JIA and JIA-associated uveitis, for example, are potentially cost-effective from a health payer perspective [7, 8]. Regulatory approval and public funding of drugs is typically dependent on support from randomized clinical trials allowing for cost-effectiveness analyses. In rare conditions, however, such data are limited or non-existent [9]. Thus, as biologic DMARDs are increasingly employed with life-changing effect, gaps in both public and private drug funding are exposed.

The World Health Organization (WHO) developed the *Model list of essential medicines* (WHO EML) “intended to meet the priority health care needs of a population” in 1977, an influential template since adapted by countries worldwide [10]. Subsequently, the WHO released a model list specifically delineating essential medicines for children (WHO EMLc) [11]. These essential medicines lists (EMLs) to guide countries’ selection of drugs to fund, stock, prescribe, and dispense [12, 13]. The Lancet Commission, “*Essential medicines for universal health coverage,*” affirms that countries “must implement a comprehensive set of policies to achieve affordable prices …” and equity in access [13]. A unique database of 138 national EMLs (71% of 195 countries) and associated country characteristics was recently compiled and demonstrates significant variation between countries in included medicines [12, 13]. We hypothesized that biologic DMARDs are underrepresented relative to conventional DMARDs in the model WHO and existing national EMLs. We therefore aimed to survey the extent to which biologic medications with primary applications in childhood inflammatory disease are included in EMLs globally, to determine country characteristics contributing to their inclusion or absence, and to contrast this with conventional DMARD therapies.

## Methods

### Data collection processes

We made use of a previously compiled database (initially constructed in June 2017, most recently updated in January 2020). To briefly summarize the initial data collection processes:

The WHO essential medicines and health products information portal, an online repository of publications of medicines and health products relevant to WHO priorities, was searched for updated versions of national EMLs. All EMLs were included irrespective of publication date and language. A data extraction method was then developed to query specific medicines within these compiled lists (from each country’s EML, medicines were manually extracted using International Nonproprietary Names). Country characteristics (WHO region; population size; life expectancy; infant mortality; gross domestic product [GDP] per capita; health care expenditure per capita; Gini index as a measure of income inequality; and the corruption perception index) were collected. Note that national EMLs include medicines for both adults and children and listing decisions may be related to total population; therefore, we collected total rather than pediatric population data. Details of the sources of these characteristics are outlined in the original publication [12].

Although the initial database construction accounted for potential redundancies in medicines (i.e. medicines considered therapeutically equivalent), this was not relevant to our analysis, as we queried only specific medicines with well-established applications in pediatric systemic inflammatory disease as below.

### Selection of medicines of interest

We sought to include all systemic biologic and conventional DMARDs in routine clinical use for the treatment of JIA, SLE, JDM, scleroderma, systemic vasculitides, and autoinflammatory disorders. We supported the selection of medicines by relevant clinical guidelines as cited below (pediatric-specific guidelines are referenced when available; note that many medicines employed in pediatric rheumatology settings are not supported by pediatric-specific clinical trials and are therefore administered “off-label”). We excluded certain novel medicines (e.g., Janus kinase [JAK] inhibitors) given their limited clinical use (Table 1).

**Table 1:** Selected medicines of interest

**Conventional DMARDs medicines (alphabetical)**	
Azathioprine [29–33]	
Calcineurin inhibitors (cyclosporine, pimecrolimus, tacrolimus considered together) [29, 34–39]	
Colchicine [40]	
Corticosteroids (cortisone, dexamethasone, hydrocortisone, methylprednisolone, prednisone, triamcinolone considered together) [29–35, 38, 39, 41–50]	
Cyclophosphamide [30–33, 36–39]	
Hydroxychloroquine [36, 37]	
Leflunomide [29, 30, 32, 34, 35, 41, 42]	
Methotrexate [29, 31–36, 38, 41–43, 45–47, 50–52]	
Mycophenolate mofetil and mycophenolate sodium considered together [29, 31–38, 44, 47, 51, 52]	
Sulfasalazine [29, 41, 42]	
**Biologic DMARDs (alphabetical)**	
Abatacept [29, 34, 35, 41, 42]	
Adalimumab [34, 35, 41, 42]	
Anakinra [39, 43, 49]	
Belimumab [36]	
Canakinumab [49]	
Certolizumab [42]	
Etanercept [34, 38, 41, 42, 49, 50]	
Golimumab [29, 35, 41, 42]	
Infliximab [29, 34, 35, 38, 39, 41, 42, 48, 50]	
Rilonacept [49]	
Rituximab [29, 31, 33, 35, 36, 41, 42, 52]	
Tocilizumab [29, 32–35, 41–43, 49]	
Ustekinumab [53, 54]	

### Data analysis

For descriptive data, we calculated medians with interquartile ranges (IQRs).

#### Comparison between countries

To determine whether country characteristics accounted for differences in medicine inclusion between countries, we created a linear regression model with the total number of included medicines as the dependent variable and the following characteristics as independent variables: WHO region, population size, life expectancy, GDP per capita, and health expenditure per capita. We fitted separate regression models for biologic and conventional DMARDs. In addition to the above variables, we included the number of conventional DMARDs on a country’s EML (“conventional DMARDs listed”) as a regressor in the analysis of biologic DMARDs. Adjusted *R*^*2*^ values for the number of independent variables are also presented. Analysis was completed using R statistical package (R Foundation, Vienna, Austria).

### Data sharing

The underlying data used in this study are publicly available and, separately, a database with updated information about national EMLs is maintained online [14].

### ethics approval

No ethics approval was sought for this review of publicly available information.

## Results

The 138 national EMLs (of 195 total countries; 71%) published between 2001 and 2017 have between 44 and 980 medicines listed (median 308, mean 366.9).

### Conventional DMARDs

We examined a total of 10 conventional DMARDs (or classes of medicines). As shown in Table 2, the most commonly listed conventional DMARD was corticosteroids, present on 100% of EMLs. Five countries (Angola, Cambodia, Djibouti, Somalia, and South Africa) list only corticosteroids and no other conventional DMARDs. Country-specific details are presented in the supplementary appendix. The least commonly listed conventional agent was leflunomide (21.74% of countries). Along with colchicine, mycophenolic acid, and sulfasalazine, leflunomide was not included in the WHO model list. The lone difference in included conventional DMARDs between the WHO EML and EMLc is the inclusion of sulfasalazine in the former. Eleven countries (7.97%) included all 10 conventional medicines of interest, while 115 (83.33%) ≥5. All countries listed at least one. The number of conventional DMARDs included ranged from 1 to 10 (median 7; IQR 5 to 8; mean 6.565).

**Table 2:** Inclusion of conventional non-biologic medicines in the WHO Model List and national essential medicines lists

Conventional non-biologic medicine of interest	Inclusion in 2019 WHO Model List of Essential Medicines (Yes/No)	Inclusion in 2019 WHO Model List of Essential Medicines for Children (Yes/No)	Total number of countries listing conventional medicine of interest (%)
**Azathioprine**	Yes	Yes	108 (78.26)
**Calcineurin Inhibitors**	Yes	Yes	91 (65.94)
**Colchicine**	No	No	88 (63.77)
**Corticosteroids**	Yes	Yes	138 (100)
**Cyclophosphamide**	Yes	Yes	115 (83.33)
**Hydroxychloroquine**	Yes	Yes	58 (42.03)
**Leflunomide**	No	No	30 (21.74)
**Methotrexate**	Yes	Yes	127 (92.03)
**Mycophenolic acid (Mycophenolate)**	No	No	55 (39.86)
**Sulfasalazine (Salazosulfapyridine)**	Yes	No	96 (69.57)

**Table 3:** Inclusion of biologic medicines in the WHO Model List and national essential medicines lists

Biologic medicine of interest	Inclusion in 2019 WHO Model List of Essential Medications (Yes/No)	Inclusion in 2019 WHO Model List of Essential Medicines for Children (Yes/No)	Total number of countries listing biologic medicine of interest (%)
**Abatacept**	No	No	4 (2.9)
**Adalimumab**	Yes	Yes	20 (14.49)
**Alemtuzumab**	No	No	12 (8.7)
**Anakinra**	No	No	5 (3.62)
**Belimumab**	No	No	2 (1.45)
**Canakinumab**	No	No	2 (1.45)
**Certolizumab**	Yes	No	8 (5.8)
**Eculizumab**	No	No	3 (2.17)
**Etanercept**	Yes	Yes	28 (20.29)
**Golimumab**	Yes	No	6 (4.35)
**Infliximab**	Yes	Yes	22 (15.94)
**Rituximab**	Yes	Yes	42 (30.43)
**Tocilizumab**	No	No	11 (7.97)
**Ustekinumab**	No	No	6 (4.35)

The multivariate linear regression indicated that the 5 included country characteristics accounted for a third of the observed differences in number of included conventional DMARDs between countries’ lists (adjusted *R*^*2*^:0.33). GDP per capita (β_1_1.02 [95% CI 0.39, 1.66]; *P* = 0.00279) was significantly associated with the total number of medicines included. Life expectancy (β_1_ 0.56 [95% CI 0.00, 1.12]; *P* = 0.05174) approached statistical significance.

### Biologic DMARDs

**Fig. 1:**
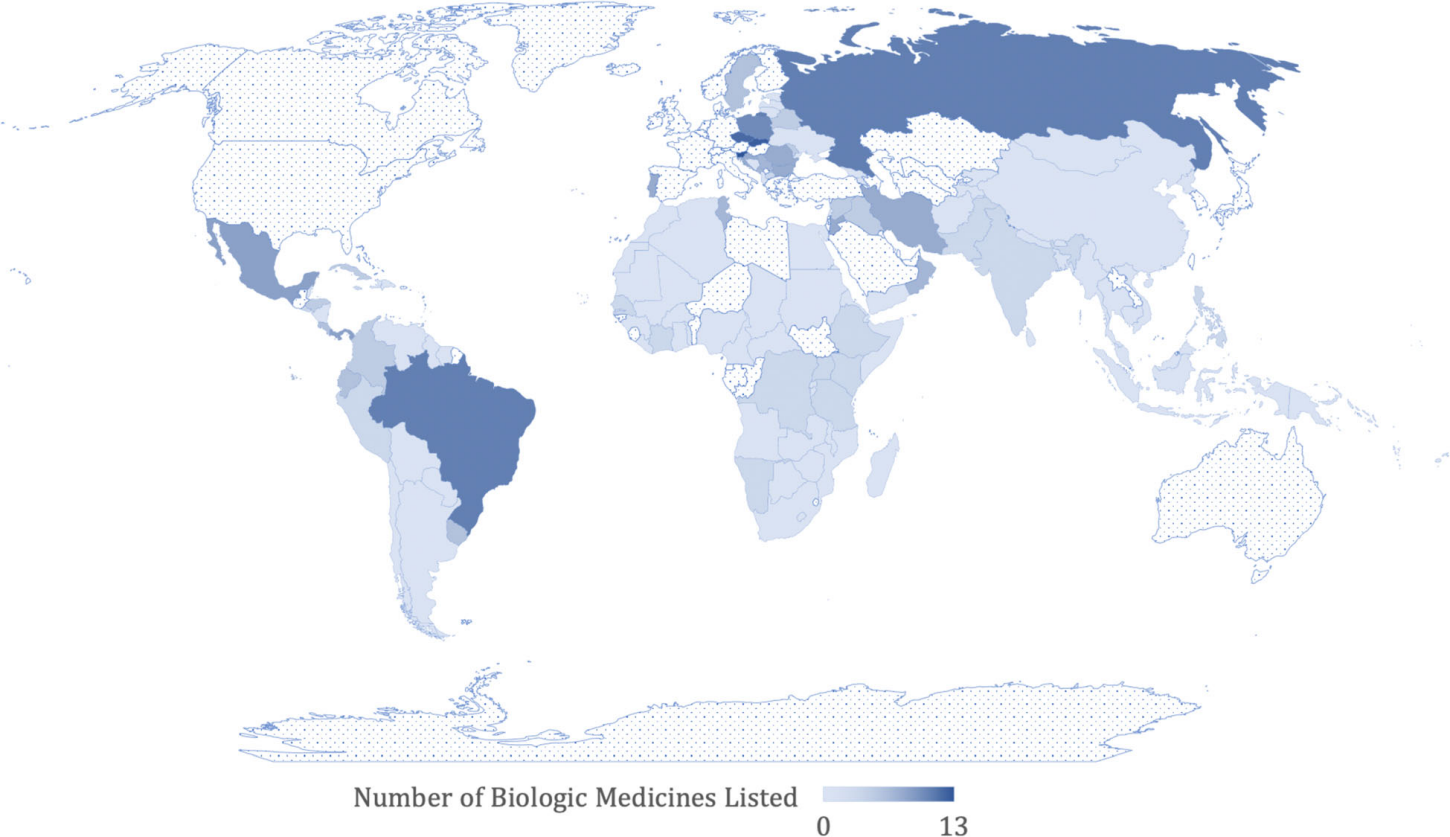
Number of biologic medicines included in national essential medicines lists by country. Notes: The number of biologic agents included ranged from 0 to 13. Countries for which no data are available are denoted by dotted pattern

We examined a total of 14 biologic DMARDs. As depicted in Table 3, the most commonly listed biologic agent was rituximab, listed by 42 countries (30.43%). Adalimumab, certolizumab, etanercept, golimumab, infliximab, and rituximab were included on the WHO EML, while only adalimumab, etanercept, infliximab, and rituximab were included on the EMLc. The least commonly listed biologics were the newer agents belimumab and canakinumab (listed by two countries [1.45%] each). As depicted in Fig. 1, Slovenia listed the greatest number of biologics at 13, while its geographic neighbors Slovakia and the Czech Republic listed 12 and 11, respectively. Thus, three countries (2.2%) listed ≥10 biologics; 15 (10.9%) were found to list ≥5, while 47 (34.1%) listed at least one biologic. Ninety-one (65.9%) of countries listed zero. The number of biologic agents included ranged from 0 to 13 (median 0; IQR 0 to 1; mean 1.239). Notably, data is unavailable for Canada and United States, among others.

**Fig. 2:**
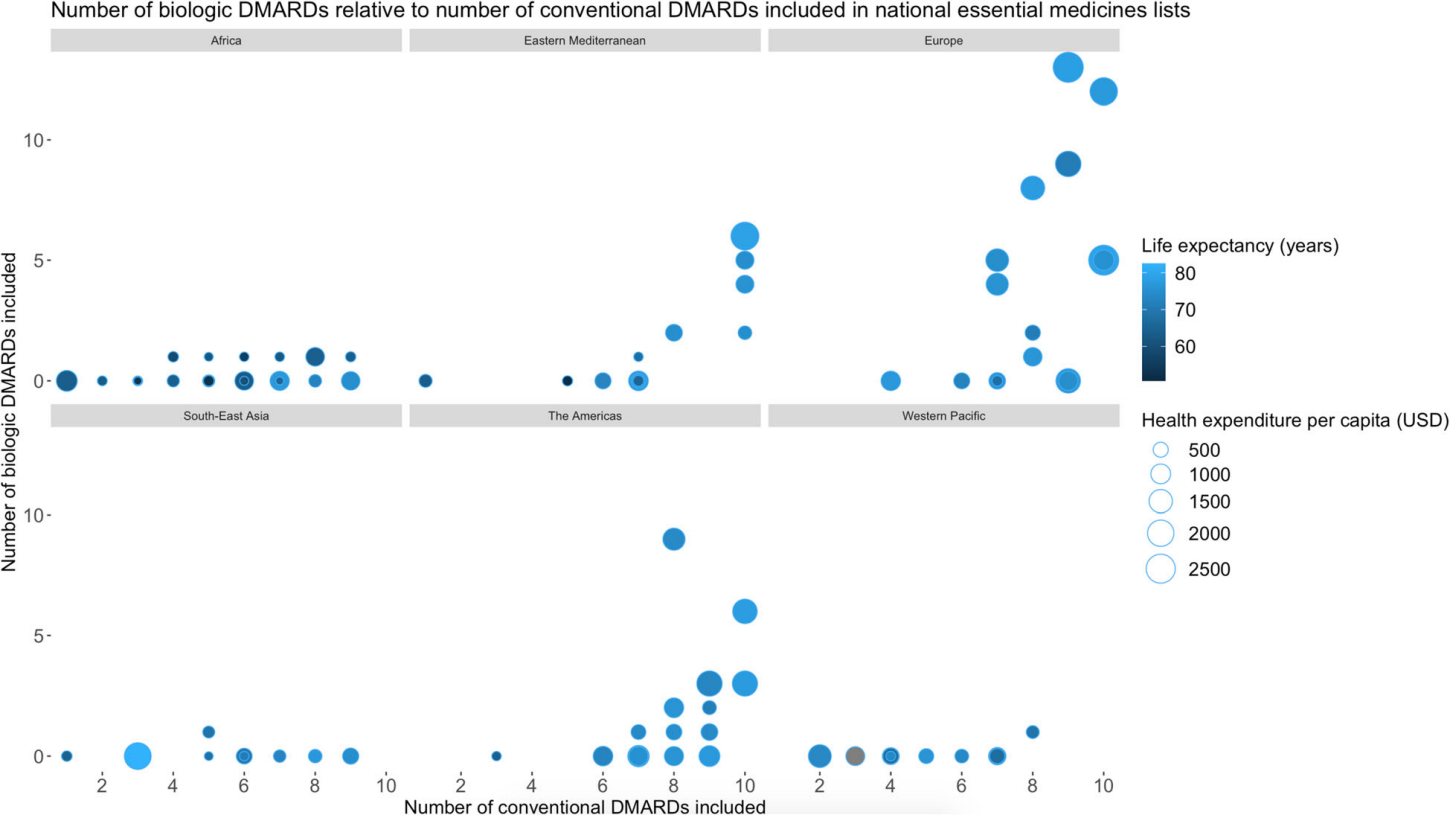
Number of biologic medicines relative to conventional medicines included in national essential medicines lists. Notes: The size of each circle represents the country’s health care expenditure per capita. The colour of each circle represents the country’s associated life expectancy. Greater life expectancy, health expenditure per capita, and number of conventional DMARDs listed are associated with a greater number of biologic DMARDs included. The association between the number of conventional DMARDs included and the number of biologic DMARDs included is visually evident in the World Health Organization (WHO) regions of Eastern Mediterranean, Europe, and The Americas

The multivariate linear regression revealed that the six included country characteristics accounted for greater than half of the observed differences in number of included biologic DMARDs between countries’ lists (adjusted *R*^*2*^:0.55).  As demonstrated in Fig. 2, European region (β_1_ 1.30 [95% CI 0.08, 2.52]; *P* = 0.0367), life expectancy (β_1_–0.70 [95% CI -1.22, − 0.18]; *P* = 0.0085), health expenditure per capita (β_1_ 1.83 [95% CI 1.24, 2.42]; *P* < 0.001), and conventional DMARDs listed (β_1_ 0.70 [95% CI 0.33, 1.07]; *P* < 0.001) were significantly associated with the total number of biologic DMARDs included, the latter two with *P* values approaching zero. The association between the number of conventional DMARDs included and the number of biologic DMARDs included is most evident in the WHO regions of Eastern Mediterranean, Europe, and The Americas.

While health expenditure per capita was predictive of biologic inclusion and GDP per capita was not, post-hoc analysis (we re-applied the regression model without health care spending per capita and found GDP to be highly statistically significant) confirmed this to be due to collinearity.

## Discussion

Biologic DMARDs with applications in childhood inflammatory disease are excluded from most national EMLs despite their potential to improve outcomes and reduce health care utilization in childhood rheumatologic illness. By comparison, conventional DMARDs, many used to treat both rheumatologic and other conditions, are widely included in national EMLs. Countries with higher health spending and longer life expectancy are more likely to list biologics. Rituximab is the most widely included biologic agent in national EMLs at greater than 30 %. Importantly, rituximab was the only biologic agent included in the 2017 version of the WHO EML; the 2019 iteration, however, introduced multiple TNF-inhibitors (adalimumab, certolizumab, etanercept, golimumab, infliximab), a reflection of the growing role of biologic therapies.

As might be expected, the two least commonly listed biologic DMARDs (canakinumab and belimumab, each listed by two countries) were included by countries with relatively expansive EMLs. Slovenia (total 13 biologics listed) included both canakinumab and belimumab, while the Czech Republic (which lists canakinumab) and Slovakia (which lists belimumab) each include 12 biologics in their respective EMLs.

To our knowledge, this is the first study to examine EML inclusion of medicines with primary applications in rare disease. This is of particular importance given their growing prevalence and associated costs to health systems [3]. The mean annual total cost of JIA, for example, is estimated between US$5683.51 (US$3637.90 in 1999) and US$50,137.91 (US$33,171 in 2000) [15]. The annualized average direct medical costs of JIA patients at two Canadian centers was found to be CAD$2119 (CAD$1686 in 2007) greater than those of healthy controls, the majority of this difference attributable to medication costs [16]. Overall, there exists a paucity of cost evidence in rare diseases—an opportunity for future research.

Strategies for EML development vary by country. In South Africa, for example, members of the National Essential Medicines List Committee (NEMLC) are appointed by the Minister of Health (MOH) on the basis of clinical, pharmacologic, public health, health economic, and bioethical expertise [17]. The process begins with an evidence-based assessment of quality, safety, and efficacy, followed by formal pharmacoeconomic evaluation. The NEMLC is the decision-making body and presents the finalized EML to the MOH for implementation [17].

Treatments for cardiovascular disease are included in most national EMLs [18]. Many drugs for HIV-AIDS that, similar to biologics, are relatively costly are now classified as essential in the WHO model lists and are commonplace on national EMLs [13]. In contrast, the WHO EML and EMLc fail to adequately address the needs of children with rheumatologic disease and do not “reflect current best practice.” [19, 20] Moreover, despite being included in the WHO model lists, the decades old and relatively inexpensive drug methotrexate—a first-line therapy for JIA—is conspicuously absent from nearly 10% of EMLs. In a recent survey of Paediatric Global Musculoskeletal Health Task Force members, five medicines were deemed “essential” for inclusion in the WHO EML (oral, intraarticular, and intravenous corticosteroids; non-steroidal anti-inflammatory drugs; hydroxychloroquine; and methotrexate), while many DMARDs—both conventional and biologic—“should” be listed [20]. Although the 2019 update of the WHO EML and EMLc partially addresses these deficiencies via the inclusion of TNF-inhibitors, further revision of the model lists is needed. Serving as influential templates for national EMLs, greater inclusion of biologics within the WHO EML and EMLc would likely improve access globally.

While a drug may be available (i.e., stocked, supplied, and dispensed) in a given country, it may not be readily accessible as a result of prohibitive costs and reimbursement policies. Access to biologic DMARDs varies between countries, resulting in discrepant health outcomes. In rheumatoid arthritis, between-country differences in GDP per capita, drug reimbursement rules, and affordability of biologics influence biologic usage and measures of disease activity, suggesting geographic inequities in access to optimal care [21]. In JIA, children living in countries with lower GDP suffer greater disease activity and damage, likely in part due to disparities in access to biologic therapies [22]. The inclusion of medications in EMLs has been shown to decrease their cost, increase availability, and improve patient outcomes over time [23, 24]. Using a model list of essential medicines for Canada, the potential savings yielded from universal public coverage of these drugs is estimated over CAD$4 billion per year for patients and private drug plan sponsors [23]. It is reasonable to extrapolate that the inclusion of biologic therapies in national EMLs and, ultimately, systems of universal prescription drug coverage would abate the economic impact and improve the quality of life of children with systemic inflammatory disease.

Our study has limitations. EML data was abstracted from the WHO website in a procedure liable to error (e.g., documents requiring translation, inconsistencies in medicine names) [12]. Next, many developed nations (namely Canada, the United States, the United Kingdom, much of Western Europe, Australia, New Zealand, Japan) do not have national EMLs. The inclusion of such high-income countries would likely not change our finding that higher health spending is associated with the listing of biologic DMARDs. Additionally, with the continued emergence of novel biologic therapies, we exluded a number of molecules with relevance in pediatric rheumatology (e.g., JAK inhibitors) from our analysis. Also, while health workforce characteristics (e.g., pediatric rheumatologists per capita) may influence medicine inclusion in EMLs, we did not have reliable workforce data for all included countries. Lastly, we note that the listing of a drug on an EML does not necessarily imply that it is available to that nation’s public; conversely, a drug may be available despite being absent from an EML. While EMLs serve to guide the supply and reimbursement of medicines, the choice of which drugs to fund, stock, prescribe, and dispense ultimately belongs to local governments, health systems, and insurers [10, 25]. Moreover, many medicines in routine clinical use for the treatment of pediatric rheumatologic conditions are administered “off-label” and their inclusion in national EMLs may be primarily motivated by alternate therapeutic indications (e.g., rituximab for hematologic malignancy) [11, 26]. The data are therefore interpreted with appropriate caution.

## Conclusion

Although biologic DMARDs are underrepresented in national EMLs and are more likely to be listed by high-income countries, this does not preclude their inclusion by less prosperous nations. For example, only nine of the 42 countries (21.4%) listing rituximab (the most commonly listed biologic) are categorized as high-income; 33 of 42 (78.6%) are therefore low- or middle-income economies [27]. This indicates the potential for other countries to consider the listing of biologic DMARDs, which would ultimately lead to a lowering of their costs and a resultant increase in their cost-effectiveness, thus rendering them more attractive to governmental and other health payers. Costs to health payers are likely to further decrease with the growth of the biosimilar market, driving price competition and improved patient access to biologic therapies [28]. The inclusion of these medicines in a system of universal prescription drug coverage would ultimately abate the economic impact and improve the quality of life of children with systemic inflammatory disease. Further study of the real-world availability and accessibility of biologic DMARDs is needed.

## Supplementary Information

**Additional file 1.**

## Data Availability

The datasets used and/or analysed during the current study are available from the corresponding author on reasonable request.

## Abbreviations

DMARD: Disease-modifying antirheumatic drug
EML: Essential medicines list
GDP: Gross domestic product
JAK: Janus kinase
JDM: Juvenile dermatomyositis
JIA: Juvenile idiopathic arthritis
SLE: Systemic lupus erythematosus
WHO: World Health Organization
