# Supplementary material for: Biologic medicine inclusion in 138 national essential medicines lists
Source: Pediatr Rheumatol Online J. 2021 Sep 6;19:140. doi: 10.1186/s12969-021-00608-z (PMC8419977; doi:10.1186/s12969-021-00608-z)
Supplement: Supplementary file 1 — Additional file 1. [file 12969_2021_608_MOESM1_ESM.docx]

|  | Conventional non-biologic DMARDs  (β_1_; 95% CI) | Biologic DMARDs  (β_1_; 95% CI) |
| --- | --- | --- |
| β_0_ | 6.42 [5.63, 7.21] *** | 0.75 [0.04, 1.47] * |
| Eastern Mediterranean | 0.66 [-0.68, 2.00] | 0.70 [-0.51, 1.92] |
| Europe | 0.41 [-0.94, 1.75] | 1.30 [0.08, 2.52] * |
| South-East Asia | -1.08 [-2.48, 0.32] | 0.27 [-1.00, 1.55] |
| The Americas | 0.42 [-0.82, 1.66] | -0.15 [-1.27, 0.97] |
| Western Pacific | -1.00 [-2.34, 0.34] | 0.20 [-1.02, 1.42] |
| Population | 0.08 [-0.26, 0.42] | 0.12 [-0.19, 0.42] |
| Life Expectancy | 0.56 [0.00, 1.12] | -0.70 [-1.22, -0.18] ** |
| GDP per capita | 1.02 [0.39, 1.66] ** | -0.32 [-0.92, 0.29] |
| Health expenditure per capita | -0.42 [-1.06, 0.23] | 1.83 [1.24, 2.42] *** |
| Conventional DMARDs listed |  | 0.70 [0.33, 1.07] *** |
| R^2^ | 0.38 | 0.59 |
| R^2^ adjusted | 0.33 | 0.55 |
| All continuous predictors are mean-centred and scaled by 1 standard deviation. *** p < 0.001; ** p < 0.01; * p < 0.05. | | |

**Supplemental Table 1.** Detailed summary of multivariate regression model results

**Supplemental Table 2.** Number of conventional DMARDs included in national essential medicines lists by country

| Country | Total conventional DMARDs included (%) |
| --- | --- |
| Afghanistan | 5 (50) |
| Albania | 7 (70) |
| Algeria | 7 (70) |
| Angola | 1 (10) |
| Antigua and Barbuda | 7 (70) |
| Argentina | 9 (90) |
| Armenia | 7 (70) |
| Bahrain | 10 (100) |
| Bangladesh | 3 (30) |
| Barbados | 9 (90) |
| Belarus | 7 (70) |
| Belize | 8 (80) |
| Bhutan | 6 (60) |
| Bolivia | 6 (60) |
| Bosnia and Herzegovina | 4 (40) |
| Botswana | 6 (60) |
| Brazil | 8 (80) |
| Bulgaria | 7 (70) |
| Burkina Faso | 5 (50) |
| Burundi | 3 (30) |
| Cambodia | 1 (10) |
| Cameroon | 5 (50) |
| Cape Verde | 8 (70) |
| Central African Republic | 5 (50) |
| Chad | 5 (50) |
| Chile | 8 (80) |
| China | 7 (70) |
| Colombia | 8 (80) |
| Congo | 8 (80) |
| Cook Islands | 5 (50) |
| Costa Rica | 10 (100) |
| Côte d'Ivoire | 4 (40) |
| Croatia | 8 (80) |
| Cuba | 8 (80) |
| Czech Republic | 10 (100) |
| Democratic Peoples Republic of Korea | 3 (30) |
| Democratic Republic of Congo | 8 (80) |
| Djibouti | 1 (10) |
| Dominica | 7 (70) |
| Dominican Republic | 7 (70) |
| Ecuador | 8 (80) |
| Egypt | 6 (60) |
| El Salvador | 8 (80) |
| Eritrea | 5 (50) |
| Estonia | 9 (90) |
| Ethiopia | 8 (80) |
| Fiji | 7 (70) |
| Gambia | 3 (30) |
| Georgia | 7 (70) |
| Ghana | 3 (30) |
| Grenada | 7 (70) |
| Guinea | 5 (50) |
| Guyana | 6 (60) |
| Haiti | 3 (30) |
| Honduras | 9 (90) |
| India | 5 (50) |
| Indonesia | 7 (70) |
| Iran (Islamic Republic of) | 9 (90) |
| Iraq | 8 (80) |
| Jamaica | 7 (70) |
| Jordan | 10 (100) |
| Kenya | 9 (90) |
| Kiribati | 6 (60) |
| Kyrgyzstan | 6 (60) |
| Latvia | 9 (90) |
| Lebanon | 7 (70) |
| Lesotho | 5 (50) |
| Liberia | 5 (50) |
| Lithuania | 9 (90) |
| Madagascar | 6 (60) |
| Malawi | 6 (60) |
| Malaysia | 9 (90) |
| Maldives | 5 (50) |
| Mali | 9 (90) |
| Malta | 9 (90) |
| Marshall Islands | 4 (40) |
| Mauritania | 2 (20) |
| Mexico | 9 (90) |
| Mongolia | 6 (60) |
| Montenegro | 7 (70) |
| Morocco | 7 (70) |
| Mozambique | 3 (30) |
| Myanmar | 6 (60) |
| Namibia | 8 (80) |
| Nauru | 7 (70) |
| Nepal | 6 (60) |
| Nicaragua | 6 (60) |
| Nigeria | 5 (50) |
| Niue | 3 (30) |
| Oman | 9 (90) |
| Pakistan | 7 (70) |
| Palau | 2 (20) |
| Panama | 10 (100) |
| Papua New Guinea | 6 (60) |
| Paraguay | 6 (60) |
| Peru | 9 (90) |
| Philippines | 8 (80) |
| Poland | 8 (80) |
| Portugal | 10 (100) |
| Republic of Moldova | 8 (80) |
| Romania | 10 (100) |
| Russian Federation | 9 (90) |
| Rwanda | 4 (40) |
| Saint Kitts and Nevis | 7 (70) |
| Saint Lucia | 7 (70) |
| Saint Vincent and the Grenadines | 7 (70) |
| Senegal | 5 (50) |
| Serbia | 7 (70) |
| Seychelles | 9 (90) |
| Slovakia | 10 (100) |
| Slovenia | 9 (90) |
| Solomon Islands | 6 (60) |
| Somalia | 1 (10) |
| South Africa | 1 (10) |
| Sri Lanka | 8 (80) |
| Sudan | 4 (40) |
| Suriname | 6 (60) |
| Sweden | 5 (50) |
| Syrian Arab Republic | 10 (100) |
| Tajikistan | 7 (70) |
| Thailand | 9 (90) |
| The former Yugoslav Republic of Macedonia | 8 (80) |
| Timor-Leste | 5 (50) |
| Togo | 7 (70) |
| Tonga | 4 (40) |
| Trinidad and Tobago | 9 (90) |
| Tunisia | 10 (100) |
| Tuvalu | 4 (40) |
| Uganda | 6 (60) |
| Ukraine | 6 (60) |
| United Republic of Tanzania | 7 (70) |
| Uruguay | 10 (100) |
| Vanuatu | 4 (40) |
| Venezuela (Bolivarian Republic of) | 8 (80) |
| Viet Nam | 6 (60) |
| Yemen | 7 (70) |
| Zambia | 5 (50) |
| Zimbabwe | 6 (60) |

**Supplemental Table 3.** Number of biologic DMARDs included in national essential medicines lists by country

| Country | Total biologic DMARDs of interest included (%) |
| --- | --- |
| Afghanistan | 0 (0) |
| Albania | 0 (0) |
| Algeria | 0 (0) |
| Angola | 0 (0) |
| Antigua and Barbuda | 0 (0) |
| Argentina | 0 (0) |
| Armenia | 0 (0) |
| Bahrain | 6 (4.35) |
| Bangladesh | 0 (0) |
| Barbados | 0 (0) |
| Belarus | 2 (1.45) |
| Belize | 0 (0) |
| Bhutan | 0 (0) |
| Bolivia | 0 (0) |
| Bosnia and Herzegovina | 0 (0) |
| Botswana | 0 (0) |
| Brazil | 9 (6.52) |
| Bulgaria | 5 (3.62) |
| Burkina Faso | 0 (0) |
| Burundi | 0 (0) |
| Cambodia | 0 (0) |
| Cameroon | 0 (0) |
| Cape Verde | 0 (0) |
| Central African Republic | 0 (0) |
| Chad | 0 (0) |
| Chile | 0 (0) |
| China | 0 (0) |
| Colombia | 2 (1.45) |
| Congo | 1 (0.72) |
| Cook Islands | 0 (0) |
| Costa Rica | 3 (2.17) |
| Côte d'Ivoire | 1 (0.72) |
| Croatia | 5 (3.62) |
| Cuba | 2 (1.45) |
| Czech Republic | 11 (7.97) |
| Democratic Peoples Republic of Korea | 0 (0) |
| Democratic Republic of Congo | 1 (0.72) |
| Djibouti | 0 (0) |
| Dominica | 0 (0) |
| Dominican Republic | 0 (0) |
| Ecuador | 3 (2.17) |
| Egypt | 0 (0) |
| El Salvador | 1 (0.72) |
| Eritrea | 0 (0) |
| Estonia | 0 (0) |
| Ethiopia | 1 (0.72) |
| Fiji | 0 (0) |
| Gambia | 0 (0) |
| Georgia | 0 (0) |
| Ghana | 0 (0) |
| Grenada | 0 (0) |
| Guinea | 0 (0) |
| Guyana | 0 (0) |
| Haiti | 0 (0) |
| Honduras | 2 (1.45) |
| India | 1 (0.72) |
| Indonesia | 0 (0) |
| Iran (Islamic Republic of) | 5 (3.62) |
| Iraq | 2 (1.45) |
| Jamaica | 1 (0.72) |
| Jordan | 5 (3.62) |
| Kenya | 1 (0.72) |
| Kiribati | 0 (0) |
| Kyrgyzstan | 0 (0) |
| Latvia | 0 (0) |
| Lebanon | 0 (0) |
| Lesotho | 0 (0) |
| Liberia | 0 (0) |
| Lithuania | 0 (0) |
| Madagascar | 0 (0) |
| Malawi | 0 (0) |
| Malaysia | 0 (0) |
| Maldives | 0 (0) |
| Mali | 0 (0) |
| Malta | 0 (0) |
| Marshall Islands | 0 (0) |
| Mauritania | 0 (0) |
| Mexico | 6 (4.35) |
| Mongolia | 0 (0) |
| Montenegro | 4 (2.9) |
| Morocco | 0 (0) |
| Mozambique | 0 (0) |
| Myanmar | 0 (0) |
| Namibia | 1 (0.72) |
| Nauru | 0 (0) |
| Nepal | 0 (0) |
| Nicaragua | 0 (0) |
| Nigeria | 0 (0) |
| Niue | 0 (0) |
| Oman | 4 (2.9) |
| Pakistan | 1 (0.72) |
| Palau | 0 (0) |
| Panama | 6 (4.35) |
| Papua New Guinea | 0 (0) |
| Paraguay | 0 (0) |
| Peru | 1 (0.72) |
| Philippines | 1 (0.72) |
| Poland | 8 (5.8) |
| Portugal | 5 (3.62) |
| Republic of Moldova | 2 (1.45) |
| Romania | 5 (3.62) |
| Russian Federation | 9 (6.52) |
| Rwanda | 0 (0) |
| Saint Kitts and Nevis | 0 (0) |
| Saint Lucia | 0 (0) |
| Saint Vincent and the Grenadines | 0 (0) |
| Senegal | 1 (0.72) |
| Serbia | 4 (2.9) |
| Seychelles | 0 (0) |
| Slovakia | 12 (8.7) |
| Slovenia | 13 (9.42) |
| Solomon Islands | 0 (0) |
| Somalia | 0 (0) |
| South Africa | 0 (0) |
| Sri Lanka | 0 (0) |
| Sudan | 0 (0) |
| Suriname | 0 (0) |
| Sweden | 3 (2.17) |
| Syrian Arab Republic | 2 (1.45) |
| Tajikistan | 0 (0) |
| Thailand | 0 (0) |
| The former Yugoslav Republic of Macedonia | 1 (0.72) |
| Timor-Leste | 0 (0) |
| Togo | 0 (0) |
| Tonga | 0 (0) |
| Trinidad and Tobago | 3 (2.17) |
| Tunisia | 4 (2.9) |
| Tuvalu | 0 (0) |
| Uganda | 1 (0.72) |
| Ukraine | 0 (0) |
| United Republic of Tanzania | 1 (0.72) |
| Uruguay | 3 (2.17) |
| Vanuatu | 0 (0) |
| Venezuela (Bolivarian Republic of) | 0 (0) |
| Viet Nam | 0 (0) |
| Yemen | 0 (0) |
| Zambia | 0 (0) |
| Zimbabwe | 0 (0) |
